# 

**Published:** 1904-04-02

**Authors:** 


### The Hospital

A JOURNAL OF

THE MEDICAL SCIENCES AND HOSPITAL ADMINISTRATION,

INCLUDING

A SPECIAL SECTION FOR NURSES.

IN TWO VOLUMES ANNUALLY.

EDITED BY

SIR HENRY BURDETT, K.C.B.,

AND

HAROLD BURROWS, M.B., F.R.C.S.

Vol. 35 incomplete

VOLUME XXXVI.

APRIL 1904 TO SEPTEMBER 1904.

SURGEON GENERAL'S OFFICE
DEC. 17 1906
190234.

London:

[PUBLISHED BY THE SCIENTIFIC PRESS (LIMITED), AT THE HOSPITAL BUILDING,]

28 & 29 SOUTHAMPTON STREET, STRAND, W.C.

MDCCCCIV.

W1 NO69X

### INDEX TO VOL. XXXVI., 1904.

⁂ Articles under the general heading *Hospital Clinics* and *Medical Progress* are distinguished by the initial (C.) for *Hospital Clinics*, and by (P.) for the rest of the series. (W. distinguishes the *Institutional Workshop* articles, and (A.) the *Annotations*.

Abdomen, Penetrating Wounds of the (C.), 98
Abscess of the Brain (P.), 363
Accidents in the Country (W.), 197
Acromegaly and Gigantism (P.), 61
Adenoid Growths of the Naso-pharynx: When and How to Operate (C.), 80
Adhesions, Perigastric (P.), 225
Adulteration made Easy (A.), 273
Albumen, Salicyl-sulphonic Test for (C.), 80
Albuminuria (P.), 227; Functional (P.), 246; in the apparently Healthy (C.), 56; Prognosis of Postural (C.), 205
Albumosuria (P.), 227
Alcohol, Cheap (A.), 257
Alcohol, Mental Effects of (C.), 371
Alcoholic Subjects, Psychical and Epileptoid Disturbances in (P.), 59
Alcoholism, Hallucinatory Tendencies in Acute (P.), 59
Alcoholism and Homicide (P.), 103; and Suicide (P.), 102
Aneurysm, Abdominal, Surgical Treatment of (C.), 222
Aneurysm of the Abdominal Aorta (P.), 345
Antrum, Distance of the, from the Cortex of the Mastoid Process (P.), 330
Anuria, Calculous (P.), 27
Apex, Fixation of the (P.), 264
Appendicitis (P.), 118, 297; (C.) 343
Appointments, 18, 34, 50, 66, 92, 108, 126, 146, 164, 180, 198, 216, 238, 254, 270, 286, 304, 320, 336, 352, 368, 414, 384, 406, 438, 454
Argyll-Robertson Pupil (P.), 391
Arsenic and the Pharmacopœia, 423
Arterio-sclerosis (P.), 315
Assam, Dispensaries of (W.), 382
Asthma, Spasmodic (P.), 347
Astigmatism and its Consequences, 20
Asylums, Private, Visits to (W.), 161, 366, 419
Auricle, Left, and Pulmonary Veins (P.), 278
Auricle, Movements of the Right (P.), 263

Bacilli, Modifications of (P.), 378
Bacteriology, Progress in (P.), 182, 247, 262, 281, 378
Balanitis, Acute, treated locally with Ethyl Chloride (C.), 292
Balfour, Mr., on the Future of the Race, 369
Barnardo's Homes, Dr. (W.), 420
Beef Tea, Note on (W.), 195
Beri-beri in the Transvaal (A.), 241
Bladder, Aniline Dyes and Tumours of the (C.), 391; Papilloma of the (P.), 43
Blood-pressure, Variations in (P.), 279
Book-keeping, Doctors and (A.), 183
Book World—
Allan. Memorandum on Infectious Diseases, for the Use of School Teachers, 30
Bagshaw and Walker. Ambulance Work, 62
Beatson. The Knights Hospitallers in Scotland, 282
Bottone. Radium, and all about it, 364
Burdett. Hospitals and Charities, 1904, 104
Caldwell. Prevention of Disease in Armies in the Field, 348
City of London Directory, 1904, 138
Clarke, J. H. Life and Work of James Compton Burnett, M.D., 158
Clarke, W. B. Meaning of a Modern Hospital, 192
Crile. Blood-pressure in Surgery, 138
Douglas. Refrigeration in the Dairy, 418
Emergency Pocket Case-book, 348
E. S. L. A Manual for the Sick and Sorrowful, 249
Fisher. Ophthalmological Anatomy, with some Illustrative Cases, 210
Francis. Asthma in Relation to the Nose, with Notes of 402 Cases, 210
Gilbey. Notes on Alcohol, 30
Glassingham. Golden Rules of Dental Surgery, 104
Head. The Food of the Gods, 46
Health Resort. May 1904, 158
Holt. Care and Feeding of Children, 332
Hutchinson, Jun. *See Treves*
Ingram and Boyle, Limited. Natural Mineral Waters; their Properties and Uses, 62
International Clinics, Vol. II., 348
Johnstone. *See Kraepelin*
Kelly's Directory of Chemists and Druggists, 1904, 192
Kirby. Practical Prescribing and Dispensing for Medical Students, 364
Kraepelin. Lectures on Clinical Psychiatry, 332
Leftwich. An Index of Symptoms as a Clue to Diagnosis, 348
Lindsay. Lectures, chiefly Clinical and Practical, on Diseases of the Lungs and Heart, 282
Lloyd. Diseases of the Appendix Vermiformis, 192
London Matriculation Directory, 418
Mann. Physiology and Pathology of the Urine, 249
Martin. A Manual of Pathology, 210
Matriculation Directory, No. XXXVI., Jan. 1904, 138
Medical Annual, 138
Mohr. *See Noorden and Mohr*
Noorden and Mohr. Acid Auto-intoxications, 348
Orthmann. Handbook of Gynæcological Pathology, 228
Owen. Medical Monograph Series, No. X. Cleft Palate and Hare Lip, 249
Paget. The Case against Anti-vivisection, 158
Paget. What we Owe to Experiments on Animals, 158
Phillips. Materia Medica, Pharmacology and Therapeutics: Inorganic Substances, 228
Pritchard. Physiological Feeding of Infants, 282
Pritchard. *See also Holt.*
Rattray. Divine Hygiene, 86
"Red Book" on Advertising, 348
Riddell. Manual of Ambulance, 332
Roberts. *See Orthmann*
Robinson. A Guide to Urine Testing for Nurses and Others, 30
Robson. Diseases of the Gall-bladder and Bile-ducts, 86
Robson. Diseases of the Stomach and their Surgical Treatment, 450
Sawyer. Contributions to Practical Medicine, 210
Sawyer. Insomnia: its Cause and Cure, 210
Schofield. Unconscious Therapeutics, or the Personality of the Physician, 364
Scientific Press, Limited. Pocket Book of Temperature Charts, 364
Souttar. Alcohol: its Place and Power in Legislation, 364
Stacpoole. Ailments of Women and Girls, 30
Sutherland. Dispensing Made Easy, 30
Symes. Bacteriology of Everyday Practice, 192
Thorne. Practical Guide to the Administration of the "Nauheim" Treatment of Chronic Diseases of the Heart in England, 62
Treves and Hutchinson. Manual of Operative Surgery, 46
Veresæff. Confessions of a Physician, 62
Vos. Easily Grown Hardy Perennials, 364
Walker. *See Bagshaw*
Whitford. Anæsthetics in Surgery, 86
Yeo. Therapeutics of Mineral Springs and Climates, 228
Younger. Insanity in Everyday Practice, 104
Bradford Union Sanatorium for Pauper Consumptives at Eastby (W.), 300
Brain, Syphilitic Disease of the (C.), 391
Breast, Predisposing and Precancerous Conditions of the (C.), 23
British Medical Association at Oxford, 325
British Science Guild (A.), 339
Brompton Hospital Report (A.), 441
Bronchiectasis (P.), 116
Bronchitis and Emphysema (P.), 102
Bulbus Arteriosus (P.), 278
Bulwer's Pathomyotomia, Dr., 434
Burma, Hospitals and Dispensaries in (W.), 89

Cairo, Anglo-American Hospital in (W.), 87
Caisson Disease and Diver's Palsy (P.), 345
Calculi, Spontaneous Fracture of Vesical (P.), 42
Calculus, Vesical, in Children, Treament of (C.), 294
Camberley Sanatorium and Convalescent Home, Heatherside (W.), 250
Cancer, Cardiac Dulness in (P.), 330; Late Marriages a possible Cause of the Increase of (P.), 44; Latest Views on (P.), 44; Progress in (P.), 9, 29, 44; Radium in (P.), 44; Research, 271; Significance of the Zoological Distribution, Nature of the Mitoses, and the Transmutability of (P.), 29; of the Tongue, Lecture on (C.), 5; Treatment of, by Injections of Sodium Cinnamate (P.), 45
Canterbury, the City Sanatorium (W.), 269
Carcinoma, Etiology of (P.), 10; of the Colon (C.), 79
Cardiosplanchnic Phenomenon (P.), 331
Carditis and Pericarditis, Rheumatic (P.), 295
Catgut, How to Sterilise (C.), 261
Catheterisation and Cystoscopy, Asepsis in (P.), 43
Cerebral, Spinal, and Nerve Surgery, Progress in (P.), 296, 312
Chamberlain at the Criterion, Mr., 256
Charity Voting Reform Association, The (A.), 37
Child-life Preservation, the Premier of New Zealand and (A.), 371
Children, Invalid, 165; Slighter Forms of Mental Defect in (P.), 156; Underfed School (A.), 387
Chloroform, Examination of the Apparatus proposed for the Quantitative Administration of (C.), 311
Chlorosis, Optic Neuritis in (C.), 185
Cholelithiasis (P.), 314
Chorea and Tænia Solium (C.), 57
Clinical Picture Galleries (A.), 95
Club Foot (P.), 119; Phelp's Operation for (P.), 119
Cocainisation, Intraspinal (C.), 115
Cold-storage in Hospitals (A.), 257
Colitis (P.), 137; Treatment of Muco-membranous (C.), 204
Colon, Carcinoma of the (P.), 248
Colonisation by the Young (A.), 289
Coma in Diabetes Insipidus (C.), 445
Complexion and Disease (A.), 371
Congress of Medicine, The 16th International, 148; (W.), 178
Consumption among Cornish Miners, 200
Cornwall as a Health Resort (A.), 3
"Costal Resistance" in Abdominal and Thoracic Diseases (C.), 133
Cough and its Significance (A.), 375
Cryoscopy (P.), 393
Cysts; Pancreatic (C.), 245; (P.), 265; of the Spleen (P.), 265
Cystoscopy (P.), 28, 415

Dacryocystitis, Treatment of (C.), 115
Death, Fear of (A.), 273
Degeneracy and Marriage, 19
Delirium, Acute (P.), 188
Dementia, Senile (P.), 174
Dental Caries and Disease (C.), 99
Dental Deterioration (C.), 446
Deptford Fund, Work of the (W.), 252
Diarrhœa, Food Poisoning and Epidemic (P.), 281
Diastolic Murmurs (C.), 151; (P.), 331
Dietetics, Progress in (P.), 85
Dionine: A New Ocular Analgesic (C.), 171
Diplococcus Pneumoniæ in Culture, Capsule Formation by (P.), 83
Diphtheria (P.), 262; of the Conjunctiva (C.), 342
Disease in Children, Society for the Study of (A.), 307
Diuretics (P.), 345
Divers and "Caisson" Disease, 93
Doses, Pharmacopœial (A.), 241
Ductus Arteriorus, Closure of the (P.), 264
Dust, Motor-cars and (A.), 409
Dysentery (P.), 137; Pathology of Asylum (C.), 97
Dysmenorrhœa, Etiology and Pathogenesis of (P.), 279

Ear Sequelæ of Acute Diseases (C.), 359
Edinburgh, Royal, Hospital for Sick Children (W.), 106; Victoria Hospital for Consumption, Craigleith (W.), 160
Editor's Letter Box, 17, 92, 125, 145, 164, 197, 237, 253, 270, 285, 352
Education of Defective Children (W.), 419
Electricity, Medical (P.), 155, 172
Empyema in Children, Bacteriology of (P.), 247
Endocarditis, Prevention of Rheumatic (C.), 243
Enemata, Hot Water Injections *vice* Nutrient (C.), 187
Epilepsy (P.), 432; Mental Automatism and Crime (P.), 136; Prognosis in (P.), 433
Epistaxis in Children, Rheumatism as a Cause of (C.), 293

Equinovarus, Congenital, during Early Infancy, Treatment of (C.), 294
Etiquette, Professional (A.), 167
Eugenics (A.), 129
Evidence in Coroners' Courts, 424
Exhibitions: Bradford, Model Hospital at the Cartwright (W.), 178; Medical, Surgical, and Hygienic (W.), 177
Exophthalmos, Pulsating (C.), 97
Expert Witnesses (A.), 273
"Extractum Belladonnæ": Interpretation of the Term, 218
Eye, Foreign Bodies in the, and their Removal with the Electro-magnet (C.), 376

Faculties, Cerebral Localisation of Mental (C.), 260
Fevers (P.), 328, 361, 377, 392, 417
Fire in Hospitals and Kindred Institutions, Precautions against (W.), 212
Food and Feeding, On (C.), 443
Foramen, Ovale, Closure of the (P.), 264
Fractures (P.), 360; Bennett's (C.), 25; Treatment of (C.), 391
Frimley, Sanatorium at (W.), 230

Gall-bladder, Progress in Surgery of the (P.), 314
Gastric Dilatation and Tetany (C.), 132
Gastric Disorders; Diagnostic Methods (P.), 117
Gastritis, Phlegmonous (P.), 117
Gastro-jejunostomy for Pyloric Stenosis, Gastric Ulcer, and some other Non-malignant Conditions (C.), 131
Genito-urinary Surgery, Progress in (P.), 8, 27, 43, 58, 134, 393, 448
Genu Varum and Genu Valgum, Surgical Pathology of (P.), 119
Giddiness (P.), 449
Girgenti Home, A (A.), 183
Glances at the Hospitals (W.), 143, 163, 320, 336, 367, 383, 396, 421, 437, 453
Glasgow District Asylum at Gartloch: Nurses' Home (W.), 435
Goître, Treatment of Exophthalmic (C.), 412
Gonorrhœa and Gonorrhœal Arthritis, Treatment of (C.), 292
Gout (P.), 26; Treatment of, Carlsbad Physician on, 128
"Goutte de Lait," The (W.), 451
Grouse Disease, 353
Growth, A Cause of Malignancy in New (P.), 29
Gynæcology (P.), 279

Hæmaturia, Some Causes of (C.), 357
Hæmoglobin, Actions of Artificial Digestive Foods on (C.), 40
Hæmophilia (P.), 11
Hæmoptysis, Treatment of (C.), 428
Hæmorrhage, Syringal, into the Spinal Cord (P.), 60
Hæmorrhages in the New-born, Intra-cranial, and Extra-cranial (C.), 115
Hampstead General Hospital (W.), 123; Infants' Hospital, Denning Road (W.), 142
Head Injuries (P.), 312
Headache, "Academy" (A.), 111; Chronic (P.), 449
Health Resort, Needs of the Modern (C.), 171
Health Resorts, British (A.), 21
Health Stations, British (W.), 64, 318
Heart, Dilatation of (P.), 295; Enlargements of (P.), 296; Irregularity of (P.), 278; Primary Non-congenital Disease of the right (P.), 330
Heart-failure in Toxæmic Conditions, Sudden (P.), 315
Heart Disease, Exercise in (P.), 331; Pathology of (A.), 95
Heart and Circulation, Diseases of the (P.), 263, 278, 295, 315, 330, 345
Hemihypertrophy of Limbs and Internal Organs (C.), 206
Hemiplegia (P.), 380; Some Cases of (C.), 427
Hernia, Congenital Origin of (P.), 173; Diaphragmatic (P.), 191; Intestinal Obstruction due to an Obturator (P.), 208; Post-operative Ventral (P.), 209; Progress in Surgery for (P.), 173, 190, 208; Properitoneal, Case of (P.), 173; Retroperitoneal, Two Cases of (P.), 190; Richter's (P.), 191; Right Duodenal, Case of (P.), 174
Herniæ of Children, Pathology and Treatment of the (C.), 389
Herpes Zoster and Facial Paralysis (P.), 61
Hip, Congenital Dislocation of the (P.), 83; Radiographs illustrating (P.), 84
Hip, Peripheral Palsies following Manual Replacement of the Congenitally Dislocated (P.), 84
Hip-joint, Tubercular Disease of the (P.), 100
Holidays and Holiday Makers, 337
Home Relief Congress, International (A.), 129
Honours, The Birthday (A.), 241
Hospitals and Opportunities, 272
Hospitals, Lord Balfour on (A.), 201
Hospitals, On Behalf of the (A.), 3
"Hospital" Library and Charities Bureau (W.), 8, 33, 107
Hospital Meetings (W.), 14, 33, 64, 90, 107, 125, 143, 161, 179, 195, 236, 253, 285, 303, 334
Hospital, Pathological Department of a Modern (C.), 25
"Hospital Penny Fund," 110
Hospital Sunday Fund (W.), 124
Hospital Sunday Sermons (W.), 215
Housing Experiment, A Glasgow (A.), 409
Housing of the Working Classes (P.), 207
How to Help our Fellows (A.), 323
Hydrocephalus, Surgical Treatment of (C.), 41
Hydronephrosis (P.), 414
Hydronephrosis, Intermittent (P.), 28
Hygienic Education, 287
Hypnotism in Chronic Alcoholism (A.), 21
Hysteria, Manifestations of (C.), 133
Hysteria, Mental State in (P.), 156

Ichthyol Compounds, Therapeutics of (C.), 187
Illness, Social Aspects of, 217
Incident, an Encouraging (A.), 201
"Index Medicus" (A.), 387
India, Hospitals and Charitable Institutions of the United Provinces of (W.), 436
Inebriate, British Institutions for the Care of the (W.), 13, 32
Inebriates, Habitual, Treatment of (A.), 3
Inebriety, Treatment of, by Atropine (C.), 278; by Atropine and the so-called "Gold Cure" (C.), 114
Infant Feeding, Citrate of Sodium in (C.), 411
Infantile Mortality, 439
Infection, Treatment of Pneumococcic (C.), 39
Infectious Disease, Spread of (W.), 159, 395
Inoculation, Anti-typhoid (P.), 392
Insane, Fragilitas Ossium in the (P.), 156; Home Care of the (A.), 219; in Workhouse Infirmaries, The, 36
Insane Poor, Care of the (A.), 53
Insanity as a Biological Problem (P.), 447; Statistics (A.), 425
Insomnia (P.), 449
Instruction in Hygiene and Temperance (A.), 323
Insufficentia Pylori (C.), 152
Intestinal Atony (P.), 137; Parasites and their Symptoms (A.), 129; Worms (P.), 137
Intestines, Progress in Surgery of the (P.), 248; Traumatic Rupture of the (P.), 249
Intravesical Separation of the Urines from the two Ureters (B.), 415

Japanese Medicine and Pharmacy (A.), 183
Joint Disease, Muscular Atrophy in (C.), 203

Kidney Disease, Functional Diagnosis of (P.), 8
Kidney Extract, Therapeutic Value of (C.), 205
Kidney, Movable (P.), 8, 394
King Edward VII.'s Hospital for Officers, 51
King's College Hospital, Festival Dinner (W.), 179
Knee-joint, Treatment of Tuberculous Disease of the (P.), 101

Labour Colonies, Lincolnshire Justices and Belgian, 12
Labyrinth, Suppuration of the (P.), 362
Lambeth Infirmary, Brook Street, New Officers' and Nurses' Home (W.), 120
Lanfine Cottage Hospital (W.), 140
Laparotomy, Closure of the Abdomen after (P.), 226
League of Mercy, Members of the, at Marlborough House (W.), 196
League of Mercy in the Colonies, A (W.), 301
Leishman-Donovan Bodies (P.), 378
Leprosy, Alleged Cure for (A.), 289
Lesions, Some Pancreatic, and their Clinical Aspects (C.), 186
Lip Reading in the Seventeenth Century, 450
Liver: Abscess, Tuberculosis of (P.), 312; Cyst of, (P.), 312; Progress in Surgery of the (P.), 314; Resection of (P.), 312
Livingstone College (W.), 214
London County Council Administration, Failure of (W.), 302
London Hospitals, Historical Sketch of Some of the (W.), 141
London Polyclinic, The (A.), 37
Lorenz's Operation (C.), 42
Lunacy and Pauper Statistics (A.), 149
Lung, Hydatid Disease of the (C.), 310
Lungs, Diseases of the (P.), 101, 116, 346; Rare Conditions (P.), 117

Maida Vale, Hospital for Epilepsy (W.), 47
Malaria (P.), 82
Malformations, Congenital, 2
Man Transformed; or the Artificial Changeling, 418
Mastoiditis (P.), 330
Measles, Some unusual complications of (C.), 188
Medical Diplomates and University Degrees, 322
Medical Education, 127
Medical Men and Hospital Abuse (A.), 425
Medical Qualifications (W.), 399
Medical Schools (W.), 399
Medical Session, the, 385
Medicine as a Profession, 386
Medicine, Tropical, Instruction in (W.), 398
Melbourne Hospital, the (W.), 452
Monifieth, N.B., Gerard Hospital (W.), 193
Meningitis, New Sign of Basilar (C.), 99
Mental Anxiety and Allied Conditions (P.), 135
Mental Disease and Leucocytic Changes in the Blood (P.), 431
Metropolitan Asylums Board: some Lost Opportunities, 354
Metropolitan Hospital (W.), 252
Metropolitan Hospital Sunday Fund (W.), 349
Middlesex Hospital, Cancer Research Department (W.), 106
Midwives Act (A.), 69
Military Surgeons and Combatant Officers, 370
Milk Supply, National, 109
Milk Supply of a Hospital: American Method of Sterilisation (W.), 284
Mistaken Identity (A.), 355
Morphine in Severe Cerebral Injuries, Prophylactic use of (C.), 260
Municipal Crêches, 338
Myopia and School Life (A.), 53
Myxœdema, Mental State in (C.) 223

Nephritis: Decapsulation of the Kidney in (C.), 41; Treatment in (P.), 246
Nerve Injuries, Treatment of Chronic (C.), 429
Nerve, Progress in, Surgery (P.), 296, 312
Nervous System, Tuberculosis of the (P.), 60
Neuralgia, Trifacial (P.), 391
Neurasthenia, The Treatment of (C.), 444; What is the Cure for? (C.), 262
Neuritis, Brachial (C.), 222; Peripheral, with Œdema (C.), 373
Neurology (P.), 379, 391, 432, 449
New Appliances and Things Medical, 29, 45, 85, 103, 157, 175, 191, 209, 227, 265, 298, 315, 331, 347, 363, 433
Notes and News, 4, 22, 38, 54, 70, 96, 112, 130, 150, 168, 184, 202, 220, 242, 258, 274, 290, 303, 324, 340, 356, 372, 388, 410, 426, 442
Nurses in the Male Wards of Public Asylums, 240
Nux Vomica Seeds, Distribution of Fat and Strychnine in (C.), 413
Nystagmus, Recognition of, 57

Oath, Asepsis and the (A.), 219
Obesity and its Treatment (C.), 131
Obituary: Adrian C. F. Hope, 124
Obstruction, Intestinal (P.), 248
Old Age (C.), 114
Operations, Abdominal, Use of Purgatives in (C.), 153
Operations, Nasal, Prevention of Sepsis in (C.), 153
Opium Poisoning, Venesection in (C.), 294
Optical Organisation (A.), 95
"Opticians" and Sight Testing, 35
Organisms in Healthy Tissues (P.), 379
Organs, Hemihypertrophy of Limbs and Internal (C.), 206
Orthopædic Surgery, Progress in (P.), 83, 100, 119
Otitis Media, Suppurative (P.), 329
Otology (P.), 329, 362
Overcrowding in the Medical Profession, 440
Oxford, New Regius Professor of Physic at (A.), 355

Pancreas, Inflammatory Affections of the (P.), 265; Progress in Surgery of the (P.), 264
Panophthalmitis, Bacteriology of (C.), 414
Paralysis, Diphtheritic (C.), 309; Facial (P.), 61; General, and Crime (P.), 446
"Paratyphoid" Fever (P.), 392
Parotitis, Secondary (C.), 221
Patent Medicines, Constituents of (A.), 111
Pelvic Floor, Operation for Repair of the (P.), 280
Pericarditis, Adherent (P.), 263
Perineal Litholopaxy (P.), 448
Peritoneum, Progress in Surgery of the (P.), 225
Peritonitis, Chronic Adhesive Sclerosing (P.), 225; Early Diagnosis of (C.), 151; Tuberculous (P.), 226
Pharmaceutical Research, Medicine and, 408
Pharmacy on the Continent (A.), 167
"Phthisis, Diffusion of," 407
Physic at Oxford, 288
Physical Characters and Morbid Proclivities (C.), 56
Physical Deterioration, 321
Physical Education in Elementary Schools, 68
Plague, Carbolic Acid and (A.), 289; Relationship of the Rat to Human (A.), 111
Plan Competitions and Difficulties of Architects: An Example (W.), 236
Pleurisy (P.), 116
Pneumonia (P.), 101, (P.), 346; Heart in Acute Lobar (C.), 244; Is Potassium Iodide a Specific in Lobar? (C.), 113; Post-Operative (C.), 204
Pneumothorax (P.), 116 (C.), 341
Poisonings in 1902, Fatal (A.), 371
Poorhouses, Management of Scotch (A.), 69
Popular Medical Lectures, 52
Post-Graduate? What is a (A.), 387
Post-Graduation Study (W.), 397
Post-Mortem Examinations in Hospitals (A.), 21
Practical Departments (W.), 65, 90, 108, 145, 303, 336, 352, 368, 381
Pregnancy, Tubal (P.), 280
Prescribe, The Right to (A.), 37
Prostate: Enlarged (C.), 294; Normal and Enlarged, Anatomy of (P.), 448; Senile Enlargement of the (P.), 134
Prostatectomy (P.), 448; Complications and Sequels of (C.), 81

19053 4

Provisions, Cost of, in Institutions (W.), 31, 49, 50, 88, 122, 139, 176, 194, 211, 229, 266, 283, 299, 333, 365
Psychiatry, Progress in (P.), 42, 59, 102, 135, 156, 174, 188, 206, 431, 447
Psychopathies, Anxious (P.), 136
Psychoses: Acute Alcoholic (P.), 42; Prodromata of the (P.), 225
Puerperal Infection, Treatment of (C.), 311
Punjab, Dispensaries and Charitable Institution of the (W.), 105
Purpura (P.), 11
Pylorus, Cancer of the (P.), 225; Congenital Hypertrophic Stenosis of the (C.), 79
Pyometra, Case of, due to Plugging of the Cervix by a Fibroid Polypus (C.), 55

Radcliffe Convalescent Hospital (W.), 105
"Remitting the Surgeon's Fee" (A.), 441
Renal Decapsulation (P.), 394
Renal Disease, Secondary Inflammations in Chronic (C.), 275
Reticence, Habit of, 306
Rheumatic Diseases (P.), 378
Rheumatism, Acute (P.), 82, 247; Acute, Abdominal Pain in (C.), 205; in Childhood (C.), 413; Chronic (P.), 11; and Gout (P.), 10, 26; Surgical or Traumatic (C.), 311
Rheumatoid Arthritis (P.), 11, 82
Road Sanitation, 147
Royal Waterloo Hospital for Children and Women (W.), 236

St. Bartholomew's Hospital (W.), 267
St. George's Hospital (W.), 232; in Danger, 255
St. Thomas's Hospital Medical School (W.), 251
Salt Tax in India, 291
Sanitarian, A Great, 305
Sanitarian, a Twentieth-Century (A.), 403
Sanitary Institute, The (W.), 302
Sanitation, International Congress on Home (W.), 215
Sarcomatosis Cutis, A Case of (P.), 45
Scarborough Hospital for Infectious Diseases (W.), 317
Scarlet Fever (P.), 393; "Return" Case of, 94
Schools, Inspection of (A.), 355
Sciatica (P.), 10
Sclerosis, Insular, Remissions and Relapses in (C.), 411
Sensory Tract (P.), 380
Serum, Antituberculous, On Treatment with (C.), 24
Shock, Effects of Alcohol, Nitro-Glycerine, and Amyl Nitrite in (C.), 153
Sialolithiasis (C.), 244
Skipping as an Exercise, 1
Small-pox (P.), 417; in Germany, 67
Smoking, Curse of Juvenile (A.), 339
Sociological Society (A.), 69
Specimens, New Method of preserving Museum (C.), 153
Specimens, Preservation of Pathological or other (C.), 55
Spine, Progress in Surgery of the (P.), 296, 312
Spleen, Progress in Surgery of the (P.), 264, 314
Standard, A Commercial (A.), 257
State Medicine (P.), 207
Sterility (P.), 280
Stomach, Diseases of the, Modern Diagnosis and Treatment of (C.), 71; Hour-glass (P.), 117; Progress in Surgery of the (P.), 224
Stomach and Intestines, Diseases of the (P.), 117, 137
Story of the Insane from Year to Year (W.), 65, 91, 144, 163, 285, 319, 382, 420, 436, 452
Students, Registration of, 199
Surgeon's Responsibilities, the, 181
Surgery, Aseptic, in Theory and Practice (P.), 344; Cerebral, Spinal and Nerve (P.), 296, 312; Genito Urinary (P.), 8, 27, 43, 58, 134, 393, 414, 448; Hernia (P.), 173, 190, 208; Intestines (P.), 248; Liver, Gall-Bladder and Spleen (P.), 314; Orthopædic (P.), 83, 100, 119; Pancreas and Spleen (P.), 264; Peritoneum (P.), 225; Progress in (P.), 360; Stomach (P.), 224; Vermiform Appendix (P.), 297
Syphilis: Aix-la-Chapelle Treatment of (C.), 358; of the Eye (C.), 343; Treatment of (C.), 291

Tabes Dorsalis, Treatment of (C.), 261
Tabes in the Negro (P.), 61; Prognosis in (P.), 380
Talipe, Paralytic, Treatment of by Tendon Transplantation (P.), 119
Taxis (P.), 209
Temperature, High, and Hygiene in Dress, 316
Tendon Jerks (C.), 259
Testicles and Vasa Deferentia, Operations on (P.), 58
Thompson, Sir Henry (A.), 53
Thyroid Grafting in Human Beings (C.), 389
Thyroidism, Acute, following Curettage (C.), 292
Tourniquets, Pneumatic (C.), 40
Tropical Diseases and Sanitation in India (A.), 307
Trusses, Inguinal (C.), 98
Trypanosomiasis (P.), 247
Tuberculosis (P.), 154, 416, 430; Early Diagnosis of (P.), 154, 416; Human and Bovine 182, (P.), 281; Laryngeal Crepitus as a Sign of Pulmonary (C.), 115; Pathogenesis of (P.), 431; Primary Muscular (C.), 293; Primary Seat of Infection in (C.), 81; Prophylaxis and Public Health (P.), 154; Royal Commission on, 166; Sanitary Treatment (P.), 430; Treatment of (P.), 189
Tumour, Cerebral (P.), 296; Intra-Cranial, a Clinical Record and Commentary (C.), 169
Tumours of the Bladder (P.), 415; of the Breast (C.), 277; of the Salivary Glands, mixed (C.), 7
Tunica Vaginalis, New Growth in the (P.), 58
Typhoid Fever (P.), 328, 361, 377; Intestinal Perforation in (P.), 249

Ulcer, Duodenal (P.), 118; Gastric (P.), 118; Gastric, Chronic (P.), 224; Gastric, Surgical Treatment of (C.), 170; Perforating Gastric and Duodenal (P.), 224
Urethra, Perineal Section for Stricture of the (P.), 44; Tuberculosis of the (P.), 58
Urethral Stricture, its Treatment and Prognosis (C.), 359
Urinary Tuberculosis (P.), 448
Urine, Pathological Conditions of (P.), 226, 246
Urology (P.), 226
Urotropin, Toxic Effects of (C.), 412
Uterus, Abscess in the Wall of (P.), 280; Inversion (P.), 280; Ventrifixation of (P.), 280

Vaccination, The Conscientious Objector and the Increase of (A.), 441
Venæ Cavæ, Closure of (P.), 263
Vermiform Appendix, Progress in Surgery of the (P.), 297
Veronal, A New Hypnotic (C.), 277
Vertigo, Aural, Treatment of (P.), 363
Veterinary Science, Degrees in (A.), 149
Victoria, Charitable Institutions of (W.), 269; Medical Charities (W.), 319
Vienna, Great New Hospital for (A.), 219
Viscera, Transposition of (C.), 223
Vision during School Life, Deterioration of (C.), 277
Vitality, A Case of Remarkable (C.), 445
Voyages d'Études Médicales (A.), 307

What the People Sleep on (A.), 323
Where Perfection Prevails (A.), 425
Women Inspectors (A.), 339
Women, Medical Schools and Medical Qualifications for (W.), 406
Women and Whisky (A), 167
Wounded, Care of the Russian (A.), 149

X-rays, Medical Uses of, and their Employment for these Purposes by Unqualified and Unskilled Persons (C.), 113

### THE SPECIAL SECTION FOR NURSES.

Aosta General Hospital, A Visit to the, 351
Appointments
Arrival of No. 1, 287
Asylum Workers' Association, 112

Bag of Hay, A, 144
Beyond the Seas: Nursing at the Anglican Mission Station, River Mambare, British New Guinea, 61
Berlin Sisters at Work, 139
Book and its Story, A, 25, 91, 142, 171, 193, 239, 278, 329, 355
British Nurses' Association Conversazione, 73

Central Midwives Board, The, 235, 252, 290; and the Examination Scheme, 208; and the Irish Training Schools, 140
Colonial Nursing Association, 153
Croydon Guardians and Sick-room Cookery, 138
Curative Treatment in Cases of Bed-sores, 99

Death in Our Ranks, 277, 314
Dinner to American Nurses, 152
Drawing-room Meeting for Nurses, 209

Echoes from The Outside World

Editor as a Patient, The, 151

Encouragement to Beginners, 59

Everybody's Opinion

Examination Questions for Nurses in the Colonies and Abroad generally, 113

Funny Experience in District Nursing, A, 63

Guy's Hospital, The Nurses' Dining Hall at, 9

How to Become a Midwife: the Rules of the New Act explained, 163, 179, 193, 207, 231, 261

International Council of Nurses, 182

Lambeth Infirmary, Opening of a Nurses' Home at, 19
Lectures on the Nursing of Infectious Diseases, 230, 260, 286, 311, 336
Lectures upon the Nursing of Mental Diseases, 18, 44, 84, 110, 136, 162, 192, 218, 246, 274, 299, 323, 348

Metropolitan Nursing Association, 88
Midwifery: as practised by Native Midwives in Fiji, 69, 86, 100
Modern Nursing of Consumption, The, 6, 32, 58, 98, 124, 150, 178, 206
My First Experience in a Boys' School, 303

National Sanatorium for Consumption, The, 337
New Nurses' Home at Gloucester, 247
Newport, Monmouthshire, An Extraordinary Incident at, 48
Night Duty Memory, A, 65
Notes on News from the Nursing World
Nurses' Bookshelf, The, 292, 303, 314
Nurses at Buckingham Palace, 250
Nurses of the Community of St. John the Divine, The, 165
Nurses' Co-operation, The: Presentation to Miss Roberts, 101
Nurses of the East London Hospital for Children, 125; of the Royal London Ophthalmic Hospital, 7
Nursing in the Army, Popularising, 33; in a Bergen Infirmary, 195; in a Boys' School, 137; of Crippled Children, The, 349; in "a Haunted Ward," 66; in an Irish Workhouse Infirmary, 324; of the Japanese Wounded, The, 85; a Patient with a Protracted Illness, 234; Questions at the International Congress of Women in Berlin, 181; Russian Soldiers wounded at Chemulpo, 45; in South Africa, 314; Tropical Diseases in London, 60
Nursing Outlook, The

Odd Leaves, 301
One Day in a Children's Convalescent Home, 87
Operation for Stone in the Bladder, 34
Oxford, The Acland Home at, 23
Plaistow Maternity Charity and District Nurses' Home, 151
Preparation for an Operation for Appendicitis, 167
Presentations
Private Nurse, The: Her Duties and How to Perform them, 300

Queen Victoria's Jubilee Institute for Nurses, 220
Question of Colour, A, 289

Reception by the Queen of the Nurses of the Royal National Pension Fund, 248
Registration of Nurses, The, 221, 232, 250
Registration of Nurses, The: Our first Debate, 46
Royal British Nurses' Association: Resignation of Treasurer, 152
Royal National Pension Fund for Nurses, 70
Rural Midwives' Association, 101

St. Bartholomew's Hospital, "View Day" at, 111
Sketches of our Hospital: A Medical Ward, 315
State Registration, 68; of Nurses, 8

Tending the Victims of the *General Slocum* Disaster, 235
"The Hospital" Convalescent Fund, 209, 265
To Nurses, 124, 237
To One of Many, 291
Trained Nurses' Annuity Fund, 73
Training of Probationers in Small Hospitals, 47
Training and Supply of Midwives, The, 127
Travel Notes and Queries

Venetian Hospital, The, 312
Victoria Hospital Bazaar, 196
Visit to an Ambulance Station in Florence, 74
Visit to the North Staffordshire Nurses' Home, 288

Ward D 3, 325
West Ham Hospital, 182
Work of County Nursing Associations, The, 263

Printed by SPOTTISWOODE & CO. Ltd., New Street Square, E.C.; and Published by the Proprietors, THE SCIENTIFIC PRESS, LIMITED, at 28 & 29 Southampton Street, Strand, London W.C.

**NOTICE TO CORRESPONDENTS.**

All MSS., Letters, Books for Review, and other matters intended for the Editor should be addressed The Editor, "The Hospital," The Hospital Buildings, 28 & 29 Southampton Street, Strand, W.C.
The Editor cannot undertake to return rejected MS., even when accompanied by stamped directed envelope.

**Notice to Advertisers and Subscribers.**

All Advertisements, Orders for Copies of The Hospital, and Business Communications should be addressed to **THE MANAGER (Not to the Editor),** The Hospital, 28 & 29 Southampton Street, Strand, London, W.C.
The Cost of Subscription, post free, is as follows:—Per week, 2½d.; per quarter, 3s. 9d.; per half-year, 7s. 6d.; per annum, 15s. Foreign and Colonial:—Per annum, 19s. 6d., payable, in all cases, in advance.

**NOTICE.—Vol. XXXIV. of "The Hospital" (April 1903 to September 1903), in handsome cloth binding, is now on sale at the office of this Journal, price 10s. 6d. Cases for binding the Numbers contained in this Volume, 2s. Index to Vol. XXXIV., 2½d., post free.**
**The Monthly part for April will be ready on April 2. Price 1s., by post, 1s. 3d.**

### Notes and News.

### THE HOSPITAL LIBRARY AND CHARITIES BUREAU.

Inquiries of the librarian are answered in this column free of charge. Inquiries to be answered promptly by post must be accompanied by a fee of 2s. 6d.

*Anon* writes:—Are ladies admissible upon the Boards of Management of hospitals?

Yes. The practice is becoming very general, especially in the case of hospitals for women and children, small hospitals, and cottage hospitals. Many hospitals have a separate ladies' committee.

### THE STUDENT OF MEDICINE.

### APPOINTMENTS.

**Charles N. Barton**, L.R.C.P.Lond., M.R.C.S.Eng., Anæsthetist to the Gordon Hospital. **Harold Barwell**, M.B.Lond., F.R.C.S.Eng., Honorary Surgeon for Throat and Ear Diseases to the Cripples' Home for Girls, Northumberland House, Marylebone. **C. C. Bullmore**, L.R.C.P.&S.Edin., L.F.P.S.Glasg., Certifying Factory Surgeon for the Falmouth District, Devonshire. **Lucy B. Clapham**, M.B.Lond., reappointed Assistant Anæsthetist, New Hospital for Women, Euston Road. **Francis G. Cross**, F.R.C.S., Divisional Surgeon for V. Division Metropolitan Police, Kingston-on-Thames and Surbiton. **Henry Devine**, M.R.C.S., L.R.C.P., Clinical Assistant to the Chelsea Hospital for Women. **T. Herbert Goodman**, M.R.C.S.Eng., L.S.A.Lond., Certifying Factory Surgeon for Haverhill and District. **Stuart Hackworth**, M.D.Lond., D.P.H.Cambs., M.R.C.S., L.R.C.P., Medical Adviser to the Hanley Education Committee. **W. Ilbert Hancock**, F.R.C.S., Assistant-Surgeon to the Central London Ophthalmic Hospital. **Ivon Hawes**, M.B., B.S.Durh., Junior House Surgeon, Royal Infirmary, Bristol. **H. D. Johns**, M.D., B.S.Durh., Certifying Factory Surgeon for the Hornsea District, Yorkshire. **Frederic W. Longhurst**, L.R.C.P.Lond., M.R.C.P.Eng., Anæsthetist to th Gordon Hospital. **R. J. Love**, L.R.C.P.&S.Edin., L.F.P.&S. Glasg., additional District Surgeon of Wodehouse, Cape Colony, Medical Officer to Indwe Land, Railway, and Collieries Company, Limited, and Medical Officer to the Indwe-Maclear Railway Construction (Cape Government Railways). **H. M. McCrea**, M.B., B.Ch R.U.I., District Medical Officer of the Wokingham Union. **J. Doig McCrindle**, M.B., C.M.Edin. Assistant Medical Officer for the Birmingham City Council. **William MacDougall**, M.A.Aberd., M.B., Ch.B.Edin., Principal Medical Officer to the Christmas Island Phosphate Company and Government Medical Officer to Christmas Island, Indian Ocean. **H. J. Orford**, M.B., Ch.B. Birmingham, M.R.C.S.,Eng., L.R.C.P.Lond., Medical Officer of Health to Klerksdorp, Transvaal, South Africa. **E. M. Pearse**, M.R.C.S.Eng., L.R.C.P.Lond., Honorary Anæsthetist, Royal Infirmary, Bristol. **Allan C. Pearson**, B.A., M.B., B.C.Cantab., M.R.C.S., L.R.C.P., D.P.H. Camb., Deputy Medical Officer, H.M. Prison, Liverpool. **W. B. Ramsden**, M.B., B.Ch.Vict., Resident Assistant Medical Officer of the Chorlton Union. **W. H. Scott**, M.R.C.S.Eng., L.R.C.P Lond., Casualty Officer, Royal Infirmary, Bristol. **G. A. C. Shipman**, M.A., M.B., B.C.Cantab., M.R.C.S., L.R.C.P., Surgeon, Grantham Hospital, Lincolnshire. **G. W. Shipman**, L.R.C.P., M.R.C.S., Honorary Consulting Medical Officer to the Grantham Hospital. **C. S. Steavenson**, M.B, Ch.B.Edin., District Medical Officer of the Darlington Union. **J. H. Stephen**, M.B., Certifying Factory Surgeon for the Banff District, Banffshire. **J. S. Townley**, M.B., Ch.B.Glasg., District Medical Officer of the Dore Union. **W. T. Webb**, M.R.C S.Eng., L.R.C.P.Lond., Resident Obstetric Officer, Royal Infirmary, Bristol.

### "The Hospital" Institutional Library.

Hospitals and Asylums of the World." 4 Vols., with a Portfolio of Plans, complete £12 12s.

"Burdett's Hospitals and Charities, 1903." 5s. net.

"Burdett's Official Nursing Directory, 1903." 8s. net.

"Cottage Hospitals, General, Fever, and Convalescent." 10s. 6d.

"Uniform System of Accounts for Hospitals and Public Institutions." New Edition. 4s. net.

"Hospital Expenditure: The Commissariat." 2s. 6d.

"A Legal Handbook for the Use of Hospital Authorities." 2s. 6d.

"The Cottage Hospital Case Book or Register of Patients." 10s. and 12s. 6d.

All these are published by the Scientific Press, Ltd., and may be obtained through any bookseller, or direct from the publishers, 28 and 29 Southampton Street, Strand, London, W.C.

